# Phosphatase and tensin homolog overexpression decreases proliferation and invasion and increases apoptosis in oral squamous cell carcinoma cells

**DOI:** 10.3892/ol.2014.2283

**Published:** 2014-06-25

**Authors:** QI GAO, LEI ZHANG, BIN ZHANG, QI-YU WANG, CHANG-FU SUN, XIAO-TING DONG, JANG YING

**Affiliations:** 1 Department of Biochemistry, Liaoning Medical University, Jinzhou, Liaoning 121001, P.R. China; 2 Department of Stomatology, The First Affiliated Hospital of Liaoning Medical University, Jinzhou, Liaoning 121001, P.R. China; 3 Department of Oral and Maxillofacial-Head and Neck Surgery, School of Stomatology, China Medical University, Shenyang, Liaoning 110002, P.R. China

**Keywords:** phosphatase and tensin homolog, invasion, apoptosis, cell proliferation, SCC-4

## Abstract

Phosphatase and tensin homolog (PTEN) is a potent tumor suppressor which regulates various cellular functions. The aim of the present study was to analyze the function of *PTEN* gene expression in squamous cell carcinoma (SCC) cells. This gene exhibits a unique function in cell migration and proliferation during the early stages of embryonic development. However, its role as a tumor suppressor gene in tongue squamous carcinoma cells remains unclear. In the present study, an SCC-4 cell line stably expressing *PTEN* was established and the effects of *PTEN* gene expression on SCC-4 cell proliferation, invasion and apoptosis were investigated. *PTEN* expression was found to induce apoptosis in SCC-4 cells, possibly via negative regulation of the phosphatidylinositide 3-kinase/Akt signaling pathway and increased expression of Bcl-2-interacting mediator of cell death. In addition, PTEN was found to control the epithelial-mesenchymal transition in SCC cells, thereby reducing their invasive ability. Furthermore, Transwell assay revealed that the expression of E-cadherin was increased, while the expression of vimentin and SNAIL was decreased. This study has provided an important insight into the mechanisms by which PTEN mediates the progression and early metastasis of tongue carcinoma.

## Introduction

Oral tongue squamous cell carcinoma (OTSCC) is one of the most common types of malignant tumors of the oral and maxillofacial region, comprising 32.3% of all cases of oral cancer. Although the pathogenesis of OTSCC remains unclear, it has been suggested that it may involve the mutation and abnormal expression of multiple genes (1). The prognosis for patients with OTSCC is relatively poor and the risk of relapse is high, which may be attributable to the highly invasive nature of OTSCC cells, the frequent movements of the tongue and the rich blood supply to the tongue. Therefore, early lymph node and late distant metastases are extremely common in tongue cancer.

With recent advancements in molecular biology, molecular genetics and related disciplines, study regarding potential treatments for OTSCC has focused on gene therapy (2). The first tumor suppressor gene with phosphatase activity identified in humans was the phosphatase and tensin homolog (*PTEN*) gene. Studies have shown that the *PTEN* gene undergoes significant mutations and deletions in a variety of tumors, including melanoma, breast, prostate and endometrial cancer, resulting in a loss of protein expression or dysfunction, thereby contributing to tumor development (3,4). Additional study has indicated that mutations and deletions in the *PTEN* gene also promote the growth and development of gliomas and head and neck cancers (5).

The epithelial-mesenchymal transition (EMT) refers to the process whereby skin-derived precursor cells undergo phenotypic changes during the embryonic and tumor progression stages. E-cadherin, a 120-kDa transmembrane glycoprotein, interacts with α-, β- and γ-catenins, as well as the E-cadherin/catenin complex, to then associate with the actin microfilament system of the cell, regulating tissue and morphological changes. Thus, the expression and functional status of the E-cadherin/catenin complex within the tumor influences cell separation and adhesion, mediating tumor invasion (6). Vimentin is often considered a marker for tumors of mesenchymal origin, and vimentin expression is increased in numerous epithelial tumors and is closely associated with tumor invasion. SNAIL is a zinc finger protein that binds to the promoter of the E-cadherin gene, inducing tumor cell EMT (7). Vimentin, SNAIL and E-cadherin are closely associated with EMT and may be useful indicators of EMT. Recent study has examined the ability of EMT to induce tumor invasion and metastasis (8); however, thus far, no reports have investigated the impact of the *PTEN* tumor suppressor gene on EMT in OTvSCC (9).

In the present study, *PTEN* was overexpressed in SCC-4 cells, and the effects of *PTEN* expression on the proliferation and apoptosis of OTSCC cells was examined. In addition, the correlation between the invasiveness of PTEN-transfected OTSCC cells and EMT-associated markers was investigated.

## Materials and methods

### Reagents and antibodies

SCC-4 cells were provided by the Ninth People’s Hospital of Shanghai Jiaotong University (Shanghai, China) and originally purchased from the Cell Bank of the Chinese Academy of Sciences (Shanghai, China). Mouse anti-human vimentin polyclonal antibodies were purchased from Santa Cruz Biotechnology, Inc. (Santa Cruz, CA, USA). Mouse anti-human E-cadherin monoclonal antibodies, rabbit anti-human Akt polyclonal antibodies, rabbit anti-phospho-Akt polyclonal antibodies and rabbit anti-human Bcl-2-interacting mediator of cell death (BIM) polyclonal antibodies were obtained from Jiamay Biotech (Beijing, China). Primer synthesis and DNA sequencing were performed by Wuhan Ying Qi Biotechnology Co., Ltd. (Wuhan, China), MTT and dimethyl sulfoxide were purchased from Promega Corporation (Madison, WI, USA), and tetramethylethylenediamine and sodium dodecyl sulfate were purchased from Sigma-Aldrich (St. Louis, MO, USA).

### Cell culture

The OTSCC SCC-4 cell line was maintained at 37°C in a humidified incubator with an atmosphere of 5% CO_2_ in Dulbecco’s modified Eagle’s medium (DMEM)/F12 medium (Hyclone; Thermo Fisher Scientific, Rockford, IL, USA) supplemented with 10% fetal bovine serum (Thermo Fisher Scientific) without any antibiotics.

### Human tissue specimens

A total of 40 human tissue specimens were collected from individuals who underwent surgery at the Department of Surgery, First Affiliated Hospital of Liaoning Medical University (Jinzhou, China) between January 2007 and December 2010. Clinical information is summarized in Table I. All patients provided written informed consent and were assessed for *PTEN* expression. This study was approved by the human ethics committee of Liaoning Medical University.

### Immunohistochemistry

**Figure 1:**
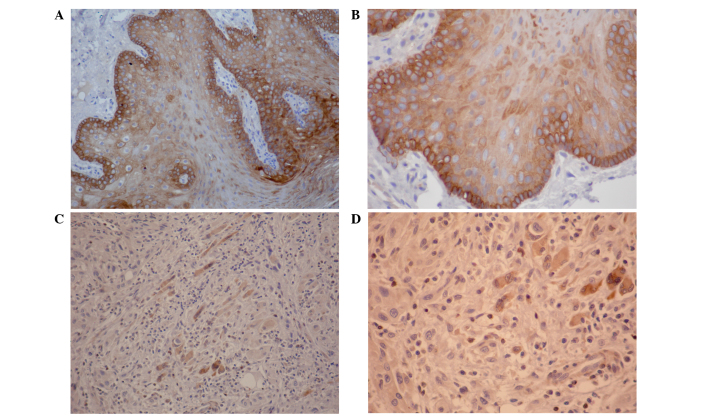
Immunohistochemical staining of PTEN in normal oral tissue and OTSCC tissue. Cytoplasmic expression of PTEN in normal oral tissue at magnifications of (A) ×200 and (B) ×400. Loss of PTEN expression in OTSCC tissue at magnifications of (C) ×200 and (D) ×400. PTEN, phosphatase and tensin homolog; OTSCC, oral tongue squamous cell carcinoma.

Immunohistochemistry was performed using a Streptavidin-Biotin Complex (SABC) kit (Wuhan Boster Biological Technology, Ltd., Wuhan, China) according to the manufacturer’s instructions. Briefly, the tissue sections were deparaffinized in xylene for 20 min and then dehydrated in graded alcohol solutions, followed by detection using the avidin-biotin complex method by SABC kit. The endogenous peroxidase activity was blocked by immersing the sections in 3% H_2_O_2_ in methanol for 30 min. For antigen retrieval, the sections were heated in 0.01 M citrate buffer (pH 6.0; Shanghai Xin Biological Technology Co., Ltd., Shanghai, China) for 15 min. The sections were then treated with 10% normal rabbit serum for 30 min, followed by incubation with mouse anti-human PTEN monoclonal antibodies [1:100 dilution; Santa Cruz Biotechnology (Shanghai) Co., Ltd., Shanghai, China] at 4°C overnight. Following incubation with a biotin-conjugated secondary antibody, incubation was performed with streptavidin solution at 37°C for 20 min, followed by incubation with SABC reagents at 37°C for 30 min. The tissues were stained with 3,3′-diaminobenzidine (Chinese sales platform ELISA kits, Shanghai, China). Negative and positive controls were conducted in each run of immunohistochemistry. A total of five to six fields from each tissue section was selection, and 100 cells from each field were counted (Countstar automated cell counter, Biomen Biosystems Co., Ltd., Guangzhou, China) at a final magnification of ×400 (Olympus BX43; Shanghai Zeshi Photoelectric Technology Co., Ltd., Shanghai, China). The evaluation was performed by two independent pathologists, without any prior knowledge of each patient’s clinical information (Fig. 1).

### Expression of PTEN mRNA by reverse transcription-polymerase chain reaction (RT-PCR)

Total RNA was extracted from SCC-4 cells using the TRIzol method (Gibco-BRL, Carlsbad, CA, USA). *PTEN* total RNA was amplified using RT-PCR. The amplification system and conditions were based on the manufacturer’s instructions stated in the Takara One-Step RNA PCR kit (Takara Bio, Inc., Shiga, Japan). Primer Premier 5.0 software (PREMIER Biosoft, Palo Alto, CA, USA) was used to design primers for the *PTEN* gene based on sequences retrieved from GenBank. The upstream and downstream primers were 5′-GCCGAATTCGACTTTTGTAATTTGTGTA-3′ and 5′-CCGCTCGAGCAGTCGCTGCAACCATCCA-3′, respectively, with *Eco*RI restriction sites introduced to the 5′ ends for nucleotide protection. For RT-PCR, each reaction was carried out as follows: Denaturation at 94°C for 5 min; 60 cycles of 94°C for 60 sec, 60°C for 60 sec and 72°C for 1.5 min; and extension at 72°C for 10 min.

### Transfection with the PTEN eukaryotic expression plasmids

SCC-4 cells growing at the logarithmic growth phase were seeded onto six-well plates and transfections were performed when the cells reached 70–80% confluence, using the Lipofectamine 2000 reagent kit (Invitrogen Life Technologies, Carlsbad, CA, USA) according to the manufacturer’s instructions. Experiments were carried out using three groups of cells: Cells transfected with phosphorylated (p)-enhanced green fluorescent protein (EGFP)-PTEN recombinant plasmid; cells transfected with pEGFP-N1 empty plasmid; and untransfected control cells. The intracellular expression of GFP was observed under a fluorescence microscope (Olympus BX43) at 24, 48 and 72 h following transfection. At 48 h following transfection, the cells were also cultured in DMEM selection medium containing 800 μg/ml G418. Cloned SCC-4 cells exhibiting stable expression of *PTEN* were then filtered for amplification and culture, and stable cell lines in the logarithmic growth phase were used for follow-up tests.

### Western blotting

Following transfection, cells were subjected to total protein extraction. Protein content was measured against bovine serum albumin, which was used as the standard. Proteins were separated by polyacrylamide gel electrophoresis on 10% gels, transferred to polyvinylidene fluoride membranes and blocked for 1 h in 5% skimmed milk. Following washing with Tris-buffered saline and Tween 20, membranes were incubated with the primary antibodies (Akt, 1:1,000; phospho-Akt, 1:1,000; BIM, 1:1,000; and PTEN, 1:500) and then incubated overnight at 4°C (PTEN) or room temperature (Akt, phospho-Akt and BIM). Next, the membranes were washed and incubated with a sheep anti-mouse polyclonal horseradish peroxidase-conjugated secondary antibody (Jiamay Biotech) for 1–2 h at room temperature. The Biospectrum imaging system (Beijing Dequan Development Trading Co., Ltd., Beijing, China) was used for image capture. The optical density of each band was measured using ImageJ software (National Institutes of Health).

### Cell proliferation assays

Following transfection, cells at the logarithmic growth phase were used for cell proliferation assays. The cell concentration was adjusted to 1×10^4^ cells/ml, and 100 μl cell suspension was added to each well of a 96-well plate (n=42 wells/cell group). Every day between day one and seven, cells were counted to measure proliferation and MTT assays were performed according to standard instructions to confirm cell viability (n=3 wells/day per cell group for each of the proliferation and MTT assays).

### Statistical analysis

All statistical analyses were performed using SPSS software, version 13.0 (SPSS, Inc., Chicago, IL, USA). Differences between all three groups were determined using analysis of variance tests, while differences between two groups were analyzed by Student’s t-test. P<0.05 was considered to indicate a statistically significant difference.

## Results

### Loss of PTEN protein expression in OTSCC

The expression of PTEN protein in OTSCC was primarily cytoplasmic, with infrequent nuclear localization (Fig. 1). PTEN expression was observed in 15 out of 15 (100%) normal oral tissues; however, loss of PTEN expression was apparent in all 25 OTSSC specimens (Fig. 1). When comparing the expression of PTEN in OTSCC with normal oral tissue, the frequency of PTEN loss was statistically significant (P<0.001). Notably, the expression of PTEN was not associated with age, gender or histological grade (data not shown).

### PTEN mRNA expression levels in each cell group

**Figure 2:**
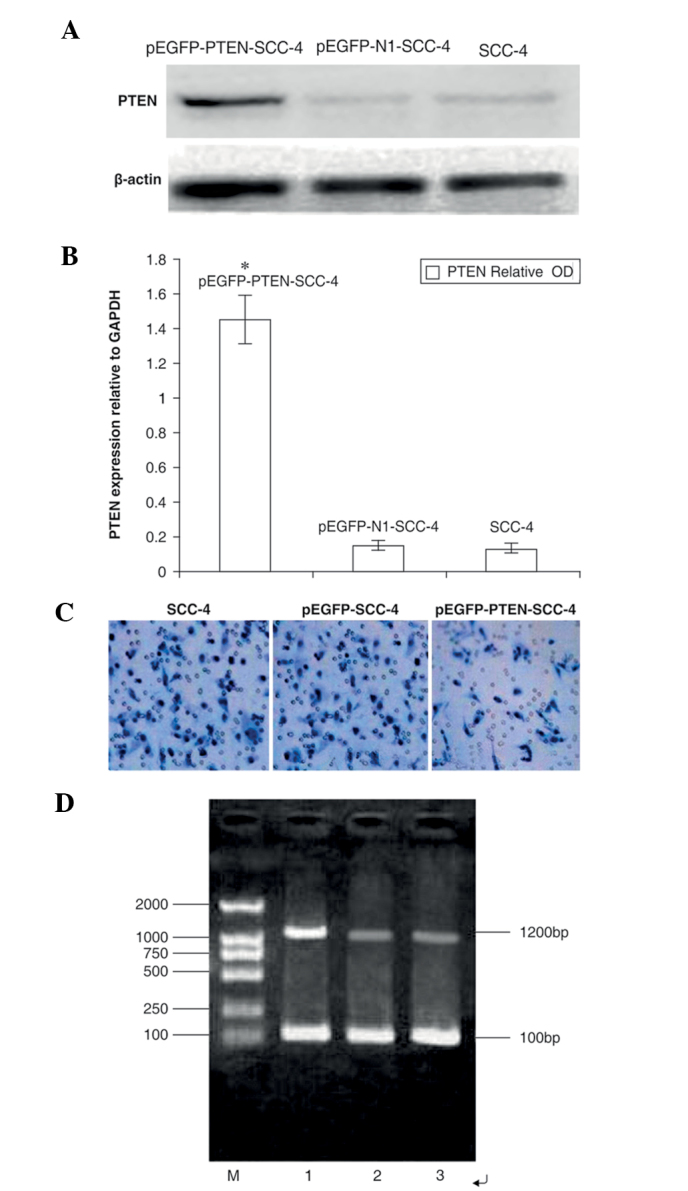
Effects of PTEN vector transfection on PTEN expression in SCC-4 cells. (A) Untransfected cells (lane 1) and cells transfected with empty vector (lane 2) or PTEN (lane 3) were subjected to western blotting using anti-PTEN antibodies. β-Actin was used as a loading control. (B) Quantification of western blots to measure PTEN protein expression. The OD of PTEN (relative to GAPDH) was as follows: 1.45±0.14, 0.17±0.02 and 0.15±0.03 in the pEGFP-PTEN-SCC-4, pEGFP-N1-SCC-4 and SCC-4 groups, respectively. (C) Effects of PTEN expression on the invasion of SCC-4 cells. Untransfected SCC-4 cells and cells transfected with pEGFP-SCC-4 or pEGPF-PTEN-SCC-4 were subjected to Transwell invasion assays. The number of cells with penetrated membranes in the pEGPF-PTEN-SCC-4 group was evidently less than in the SCC-4 and pEGFP-SCC-4 groups. (D) Reverse transcription-polymerase chain reaction for PTEN gene mRNA expression. M, DNA maker; 1, pEGFP-PTEN-SCC-4; 2, pEGFP-N1-SCC-4; and 3, SCC-4. PTEN, phosphatase and tensin homolog; pEGFP, phosphorylated enhanced green fluorescent protein; SCC, squamous cell carcinoma; OD, optical density.

The results of 1% agarose gel electrophoresis revealed that the three groups of cells appeared in ~1,200-bp bands. However, the pEGFP-PTEN-SCC-4 group was found to exhibit significantly thicker bands (Fig. 2D).

### PTEN expression in SCC-4 cells

Western blotting results revealed that the transfected group exhibited a significantly increased brightness of bands when compared with the other groups. The optical density of PTEN protein expression (relative to GAPDH) in PTEN-transfected cells was 1.07±0.15, which was identified to be significantly different when compared with that of cells transfected with the empty vector and untransfected cells (0.62±0.11 and 0.57±0.08, respectively; P<0.05; Fig. 2A and B). These results indicated that transfection with the PTEN-containing plasmid induced overexpression of exogenous PTEN.

### PTEN overexpression suppresses SCC-4 cell proliferation

**Figure 3:**
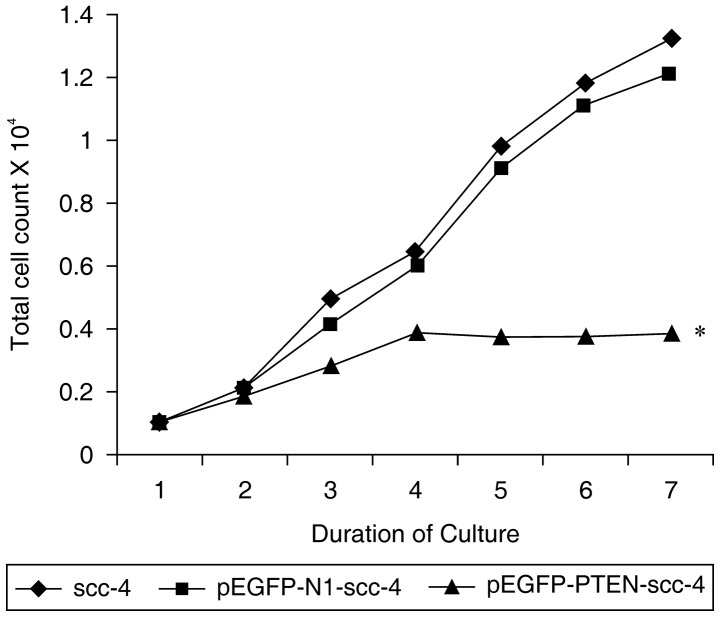
PTEN expression suppresses SCC-4 cell proliferation. Cell proliferation was measured by counting the number of cells each day for seven days PTEN, phosphatase and tensin homolog; SCC, squamous cell carcinoma, pEGFP, phosphorylated enhanced green fluorescent protein.

The effects of PTEN overexpression on SCC-4 cell proliferation were investigated. Notably, transfection with the pEGFP-PTEN-SCC-4 vector resulted in a significant reduction in cell proliferation when compared with that of the pEGFP-N1-SCC-4 and SCC-4 groups following the third day of culture (P<0.01; Fig. 3). These results indicated that PTEN expression suppresses SCC-4 cell proliferation.

### Overexpression of PTEN induces apoptosis in SCC-4 cells

**Figure 4:**
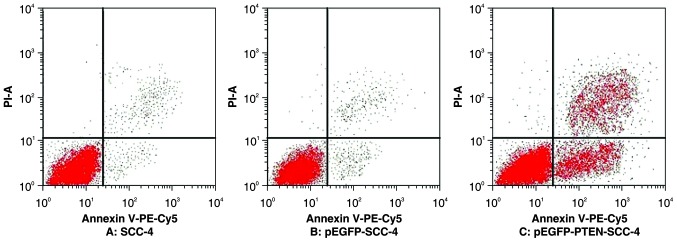
Analysis of apoptosis in SCC-4 cells by flow cytometry. At 48 h following transfection, cells were stained with Annexin V-PE-Cy5/PI and subjected to flow cytometry analysis. SCC, squamous cell carcinoma; PE, phycoerythrin; PI, propidium iodide; pEGFP, phosphorylated green fluorescent protein; PTEN, phosphatase and tensin homolog.

Flow cytometry analysis of Annexin V-phycoerythrin-Cy5/propidium iodide double-staining indicated that the percentage of apoptotic cells was significantly higher in PTEN-GFP-transfected cells when compared with that of cells transfected with the empty vector and untransfected control cells (48.1±2.6, 1.2±0.7 and 1.4±0.9%, respectively) 48 h following transfection. No significant differences in apoptotic rate between the control and empty vector groups were identified (t=0.3; P>0.05). However, a significant difference was identified between the apoptotic rate of the experimental group and that of the other two groups (t1=30.2; t2=29.4; P<0.01) (Fig. 4).

### Effects of PTEN expression on Akt, phospho-Akt and BIM levels in tongue cancer

**Figure 5:**
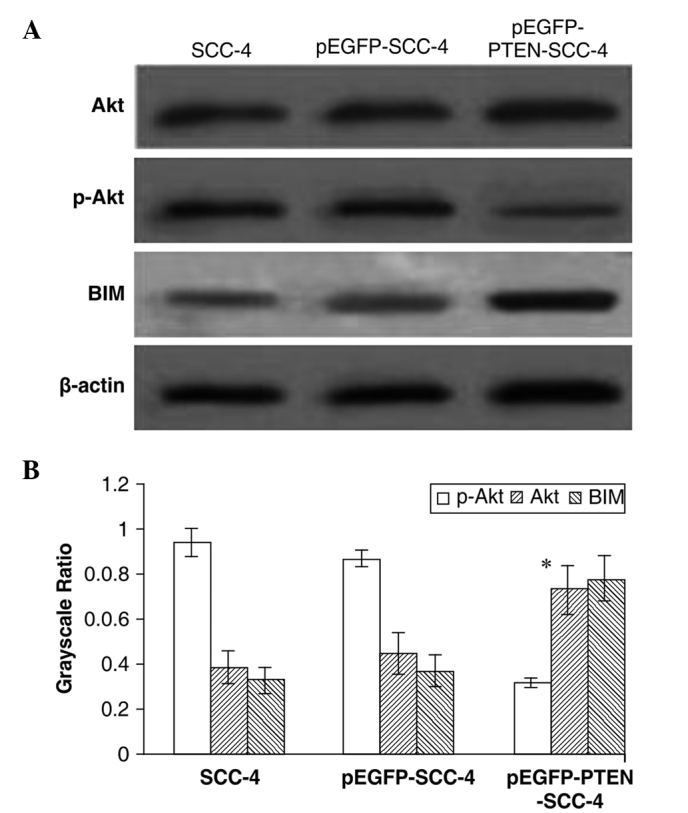
(A) Effects of PTEN expression on Akt, phospho-Akt and BIM levels in SCC-4 cells. Untransfected cells (lane 1) and cells transfected with empty vector (lane 2) or PTEN (lane 3) were subjected to western blotting using the indicated antibodies. β-actin was used as a loading control. (B) Quantification of phospho-Akt, Akt and BIM protein expression. PTEN, phosphatase and tensin homolog; BIM, Bcl-2-interacting mediator of cell death; pEGFP, phosphorylated enhanced green fluorescent protein; SCC, squamous cell carcinoma.

No significant differences in total Akt expression were identified among transfected and untransfected cells following western blotting. However, while phospho-Akt levels were 0.94±0.13 for SCC-4, 0.87±0.04 for pEGFP-SCC-4 and 0.32±0.02 for pEGFP-PTEN-SCC-4 (P>0.05), while phospho-Akt levels were significantly reduced in PTEN-transfected cells (P<0.05). The expression of BIM, a pro-apoptotic, BH3-only protein member of the Bcl-2 family which is critical in apoptosis (10), was 0.33±0.06 for SCC-4, 0.37±0.07 for pEGFP-SCC-4 and 0.78±0.10 for pEGFP-PTEN-SCC-4; however, it was found to be significantly upregulated in PTEN-transfected cells (P<0.01) (Fig. 5).

### Overexpression of PTEN inhibits SCC-4 cell invasion

**Figure 6:**
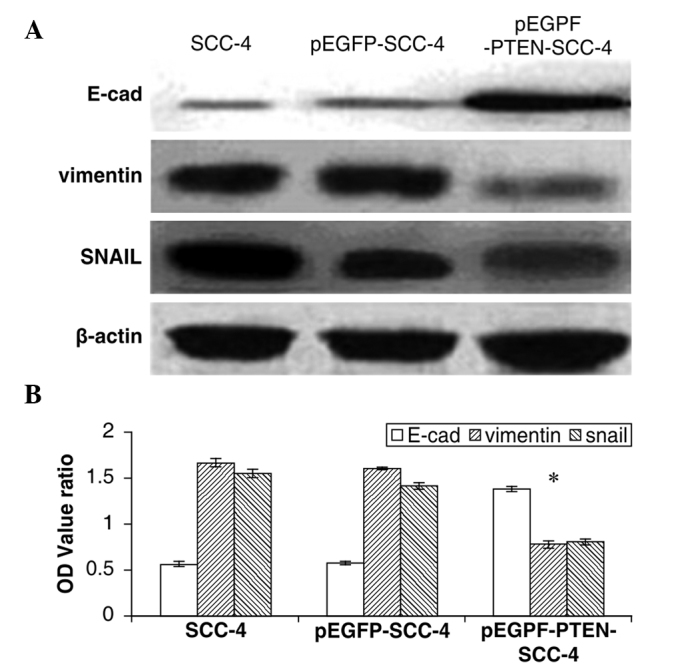
(A) Effects of PTEN expression on E-cadherin, SNAIL and vimentin proteins levels in SCC-4 cells. Untransfected cells (lane 1) and cells transfected with empty vector (lane 2) or PTEN (lane 3) were subjected to western blotting using the indicated antibodies. β-Actin was used as a loading control. (B) Quantification of E-cadherin, SNAIL and vimentin protein expression. PTEN, phosphatase and tensin homolog; SCC, squamous cell carcinoma; pEGFP, phosphorylated enhanced green fluorescent protein; OD, optical density.

Cells in the control group (SCC-4), empty vector group (pEGFP-SCC-4) and transfected group (pEGFP-PTEN-SCC-4) were cultured in Transwell invasion chambers for 36 h. For the 30 visually selected fields from each group, the numbers of cells invading through the membrane were 82±5, 80±4 and 42±5, respectively (Fig. 2C). These results demonstrated that PTEN expression caused a significant reduction in SCC-4 invasion when compared with the control (P<0.01). In addition, a statistically significant difference was identified when comparing the expression of the three proteins in the transfected and control groups (P<0.05). However, no significant differences were identified between the protein expression of the empty vector and control groups (P>0.05). The results of the western blotting revealed that PTEN expression significantly induced the expression of E-cadherin (SCC-4, 0.556±0.022; pEGFP-SCC-4, 0.573±0.013; and pEGFP-PTEN-SCC-4, 1.375±0.026) and suppressed the expression of SNAIL (SCC-4, 1.554±0.041; pEGFP-SCC-4, 1.412±0.036; and pEGFP-PTEN-SCC-4, 0.801±0.027) and vimentin (SCC-4, 1.667±0.045; pEGFP-SCC-4, 1.593±0.013; and pEGFP-PTEN-SCC-4, 0.778±0.032) (P<0.01) (Fig. 6). These results suggested that PTEN may block OTSCC cell invasion by inhibiting the EMT process.

## Discussion

*PTEN* is a tumor suppressor gene with dual phosphatase activity. However, its mechanism of action is not fully understood (11). At present, PTEN is considered to convert dephosphorylated phosphatidylinositol (3,4,5)-triphosphate (PIP_3_) to phosphtidylinositol (4,5)-bisphosphate, thereby blocking PIP_3_-mediated activation of protein kinase B/Akt and suppressing the growth and development of tumors (12). In addition, PTEN has been shown to function in the nucleus and thus may be important in transcriptional regulation, however, its nuclear targets remain unclear (13).

In the present study, PTEN expression was detected in OTSCC specimens and the effects of PTEN expression in SCC-4 cells transfected with a PTEN expression vector were investigated. Using this model, PTEN expression was found to exert a tumor suppressor function, which significantly reduced the proliferation capacity of SCC-4 cells, thus confirming the function of PTEN in the malignant behavior of OTSCC.

Deletion of the *BIM* gene may lead to tumorigenesis (14). Previous studies have shown that numerous anticancer drugs, including those for lung (15) and ovarian (12) cancer, induce tumor cell apoptosis via the increased expression of BIM. In the current study, the results of the western blotting indicated that BIM expression increased following transfection with a *PTEN* expression vector in SCC-4 cells, suggesting that PTEN expression may affect the phosphatidylinositide 3-kinase (PI3K)/Akt signaling pathways via upregulation of BIM. The results of this study also confirmed that it is possible to induce apoptosis of SCC-4 cells via *in vitro* PTEN transfection, possibly through negative regulation of the PI3K/Akt signaling pathway and increased expression of the transcription factor Akt and the pro-apoptotic protein BIM, thereby enhancing SCC-4 cell apoptosis. Since the PI3K/Akt pathway involves a variety of additional factors, further targets for gene therapy are available.

It has been demonstrated that PTEN suppresses tumor development by promoting apoptosis of tumor cells and regulating the cell cycle, reducing the invasiveness of tumor cells in esophageal cancer and melanoma (16). Similarly, in the current study, PTEN expression was reduced in advanced tumors and tumors undergoing lymph node metastasis.

Loss of E-cadherin and upregulation of vimentin are hallmarks of the EMT. In addition, E-cadherin^low^/vimentin may be used as an indicator of tumor prognosis, whereby a small ratio indicates poor prognosis (18). Furthermore, the transcription factor SNAIL is important in EMT. Häyry *et al* (19) revealed that in 73 cases of OTSCC, SNAIL expression and depth of invasion were found to significantly correlate, demonstrating that SNAIL directly affects tumor invasion and metastasis. Thus, the differential expression of these three proteins in OTSCC cells indicates the varying degrees of EMT. In addition to the results of the current study regarding cell invasion, these results show that PTEN regulates the expression of E-cadherin vimentin and SNAIL, indicating the involvement of PTEN in EMT. Thus, when considering the invasive abilities of the different groups of cells, we hypothesize that the invasive ability of OTSCC cells is associated with the EMT process.

*PTEN* gene deletion induces EMT via the PI3K/Akt signaling pathway (20), thereby increasing the invasive ability of tumor cells. Additionally, Leslie *et al* (21) reported that colorectal cancer cells become spindle-shaped following treatment with LY294002, a PI3K/Akt-specific inhibitor, an effect which was accompanied by a reduced expression of E-cadherin and increased invasiveness of the cells, further supporting the role of the PTEN/E-cadherin signaling axis in EMT.

In conclusion, the PTEN gene is closely associated with the development of SCC. Expression of the *PTEN* gene may inhibit the growth of tongue SCC cells. This may present a possible line of gene therapy. However, OTSCC is a solid tumor and its structure and biological characteristics are extremely complex. Therefore, the SCC-4 cell line does not fully reflect the tumor itself and cannot present the complete genetic characteristics of OTSCC. Thus, PTEN must be studied using animal models to elucidate its detailed mechanism of action.

## Figures and Tables

**Table I:** Clinicopathological features of OTSCC.

Clinicomorphological parameters	n (%)
Age, years	
<20	6 (15.0)
20–40	14 (35.0)
>40	20 (50.0)
Gender	
Female	26 (65.0)
Male	14 (35.0)
Normal oral tissues^a^	
Female	10 (66.7)
Male	5 (33.4)
OTSCC tissues	
Female	18 (72.0)
Male	7 (28.0)

a Obtained from healthy volunteers.

OTSCC, oral tongue squamous cell carcinoma.
